# Comparative analysis of combined exercise programs in middle-aged obese males: impact on energy expenditure, body composition, and metabolic rate

**DOI:** 10.3389/fspor.2025.1533030

**Published:** 2025-04-01

**Authors:** Gerasimos V. Grivas

**Affiliations:** Department of Physical Education and Sport Science, University of Thessaly, Trikala, Greece

**Keywords:** energy expenditure, concurrent training, exercise, obesity, physical fitness

## Abstract

**Introduction:**

This study compares energy expenditure, body composition, and metabolic rate between serial (SCG) and integrated (ICG) combined training in untrained middle-aged obese males. Method: Twenty untrained obese males (age: 50 ± 3 years) were randomly assigned to a SCG (*n* = 10) or an ICG (*n* = 10). Both groups completed a 3-month training program (3 sessions/week) consisting of walking and bodyweight exercises, differing only in the sequence of aerobic and strength training. In SCG, strength training was performed before aerobic training, while in ICG, aerobic and strength exercises alternated in a predetermined order.

**Results:**

After a 3-month training period, both groups significantly improved all health indices compared to pre-training values (*p* < 0.005, *g* = 0.40–2.71), indicating small to large effects. Specifically, there were moderate reductions in body fat percentage, small decreases in body circumferences, and moderate-to-large reductions in arterial blood pressure and resting heart rate. Additionally, respiratory function showed a large improvement. No significant differences were observed between SCG and ICG in energy expenditure, resting metabolic rate, total daily energy expenditure, macronutrient composition, or health indices after the intervention (*p* > 0.05).

**Conclusion:**

In conclusion, both SCG and ICG are effective strategies for improving metabolic health, respiratory function, and body composition in middle-aged obese males. These findings highlight the flexibility of combined training approaches in promoting overall health and fitness in this population and suggest that both combined training programs can be implemented in structured exercise programs to promote cardiometabolic health in middle-aged adults.

## 1 Introduction

Physical inactivity increases the risk of cardiovascular diseases and the other chronic diseases as obesity, diabetes, hypertension and cancer (1, 2). Obesity, affecting millions worldwide, particularly children and adolescents, is a significant global health issue (3). To prevent obesity and other chronic diseases, the World Health Organization (4) recommends that untrained healthy adults engage in regular physical activity 3–5 times per week (5). Walking, in particular, is a popular, convenient, and cost-free form of aerobic exercise that can be easily integrated into daily routines and maintained throughout life (6). Numerous studies demonstrate the beneficial effects of walking on aerobic capacity, lipid profile, body composition, and blood pressure (7–9). In addition to aerobic activities like walking, incorporating strength training into the program can enhance health-related benefits (10). For this reason, over the past decades, numerous researchers have recommended designing and implementing various combined training programs that include both cardiorespiratory and neuromuscular exercises. These programs aim to improve overall health, functional capacity, and physical fitness parameters (10).

There are two forms of combined training programs: the serial method, where strength training is completed before or after aerobic exercise in each session, and the integrated method, where aerobic and strength routines are alternated repeatedly during the training session (1). Although numerous studies have examined the effects of serial combined aerobic (walking) and strength training programs on physical fitness and health indices (11–15), fewer studies have investigated integrated combined training programs. Additionally, while some research has directly compared serial combined training program (SCG) and integrated combined training program (ICG), these studies have primarily focused on different populations, such as trained individuals, female participants, or athletes. To our knowledge, only one study has directly compared the efficiency of SCG and ICG aerobic (walking) and strength training programs in middle-aged overweight untrained men (1). In this study, both combined training programs improved health indices and physical fitness, with no significant differences between them (1).

Various studies have compared the energy expenditure of healthy adults using newer aerobic devices with traditional forms of aerobic exercises, such as walking, running, or cycling (16, 17). Many studies have also compared the energy expenditure of modified forms of walking with regular walking (18–20). However, differences in energy expenditure between SCG and ICG may be influenced by their distinct physiological demands. The structured nature of SCG may allow for greater total work output in each exercise mode, while the intermittent alternation of aerobic and strength exercises in ICG may elicit greater cardiovascular and metabolic responses, potentially leading to differences in fat oxidation, oxygen consumption, and recovery dynamics. For example, Church et al. (20) compared regular walking with Nordic walking and found that Nordic walking induced significantly higher energy expenditure than regular walking. However, few studies compare the energy expenditure of training regimes that involve resistance training (21). Therefore, only a few studies have compared the energy expenditure using SCG or ICG aerobic and strength training programs. Hunter et al. (22) compared the energy expenditure of a combined aerobic and strength training program with that of aerobic training or strength training alone in untrained older women. They found that only the combined training program significantly decreased energy expenditure (−150 kcal/day). Similarly, Pichon et al. (23) compared the energy expenditure of traditional resistance training and circuit weight training in young adults. The results of this study suggest that the circuit training program induced higher energy expenditure than traditional resistance training. In another study, Monteiro et al. (24) compared the energy expenditure of circuit weight training with that of compounded circuit training (an integrated training program). The ICG induced higher energy expenditure than the typical circuit training. Additionally, no previous study has compared the energy expenditure of two outdoor combined exercise programs (serial and integrated) in obese middle aged untrained men.

In recent years, the measurement of energy expenditure using the latest sport watches has become increasingly prevalent. Therefore, a study by Roos et al. (25) estimated energy expenditure during aerobic running using three different sport watches. They found that the Polar V800 (the same watch used in this study) is recommended for accurate assessment of energy expenditure.

However, to our knowledge, only three studies (1, 26–29) directly compared the effectiveness of SCG and ICG reported equivocal findings. Grivas et al. (1) showed that SCG and ICG aerobic (walking) and strength training (body weight exercises) led to similar benefits in middle-aged males. Similarly, Karatrantou et al. (29) observed that SCG and ICG aerobic (aerobic dance) and strength training (body weight exercises) led to similar benefits in middle-aged females. On the other hand, Davis et al. (26–28) reported that ICG aerobic (treadmill running) and strength training (resistance exercises) was slightly superior in improving some fitness parameters in young collegiate track and field athletes. However, there is evidence that subjects' characteristics (e.g., age, sex) and physical fitness level are associated with different cardiovascular responses, peripheral fatigue development, and substrate utilization during exercise (30, 31), potentially leading to varying neuromuscular and aerobic training adaptations (32, 33). Additionally, the mode of aerobic exercise in a combined training program may influence the extent of neuromuscular adaptations (34).

Thus, the main objectives of this study were: (a) to examine and compare the energy expenditure, resting metabolic rate, total daily energy expenditure and macronutrient composition of SCG and ICG, and (b) to assess and compare the efficacy of SCG and ICG on health physical fitness indices.

## 2 Methods

### 2.1 Participants

The study participants were 20 healthy, untrained obese men aged between 44 and 54 years old. Obesity in this study was determined based on body mass index (BMI), body fat percentage, and waist-to-hip ratio, following the World Health Organization (WHO) and American College of Sports Medicine (ACSM) guidelines. Participants were classified as obese if they had a BMI ≥30 kg/m^2^, a BF% ≥25%, or a waist-to-hip ratio >0.90 for males. These criteria ensured that participants exhibited both general and central obesity, making them an appropriate population for investigating the effects of combined training modalities on metabolic and physiological adaptations. The sample size was determined based on the convenience. Prior to the study, participants' health (health history questionnaire, resting electrocardiogram, and echocardiogram examined by a cardiologist) and activity status were assessed. Their activity status was assessed using a structured questionnaire, which included questions regarding the frequency, duration, and intensity of physical activity over the past year. The questionnaire specifically evaluated participation in structured strength or endurance training programs, as well as occupational and recreational physical activity levels, to ensure that participants met the criteria for inactivity. Additionally, verbal confirmation of their inactivity status was obtained during an initial interview conducted by the research team. All participants met the following criteria: (1) inactive status (i.e., without systematic strength or endurance training for at least one year before the study), (2) free from chronic illnesses, and (3) no injuries or diseases. Participants were thoroughly informed about the potential risks, discomforts, and benefits of the study. Subsequently, they received detailed information about the study design, measurements, and procedures and provided written informed consent form. Participants were fully informed about the study's hypotheses and objectives as part of the informed consent process, as the study relied on objective physiological and metabolic measurements rather than self-reported outcomes. The procedures were following the Helsinki declaration of 1975, as revised in 2000, and approval was received from the Ethics Committee of the local university.

### 2.2 Study design

Two weeks before the beginning of the training protocol, subjects completed a laboratory familiarization session and were informed about the proper form of exercises. Thereafter, indices of health were assessed on 2 separate days, with a 24 h interval between sessions to minimize participant fatigue and ensure accurate data collection. Two days following the pre-training testing, the subjects were randomly divided into two equal groups (*n* = 10 per group): SCG and ICG (Table 1). During the study, SCG and ICG participants were involved in a 3-month outdoor combined training program. All measurements were repeated after 3-months of training. The measurements were made at the same time of the day to minimize the effects of circadian fluctuation and started two days after the end of the training intervention. Before testing, all participants were asked to refrain from: (a) alcohol or caffeine consumption within 3 h of testing, (b) smoking within 3 h of testing, and (c) strenuous physical strength and endurance activities within 48 h of testing.

**Table 1:** Subjects characteristics per group (values are means ± SD).

Variables	SCG (*n* = 10)	ICG (*n* = 10)
Age (years)	51 ± 3	49 ± 3
Body mass (kg)	99.10 ± 21.23	96.20 ± 18.02
Body height (m)	1.78 ± 0.06	1.76 ± 0.04
BMI (kg/m^2^)	31.27 ± 5.31	30.88 ± 4.69

SCG, serial combined group; ICG, integrated combined group; BMI, body mass index.

### 2.3 Training program

**Figure 1:**
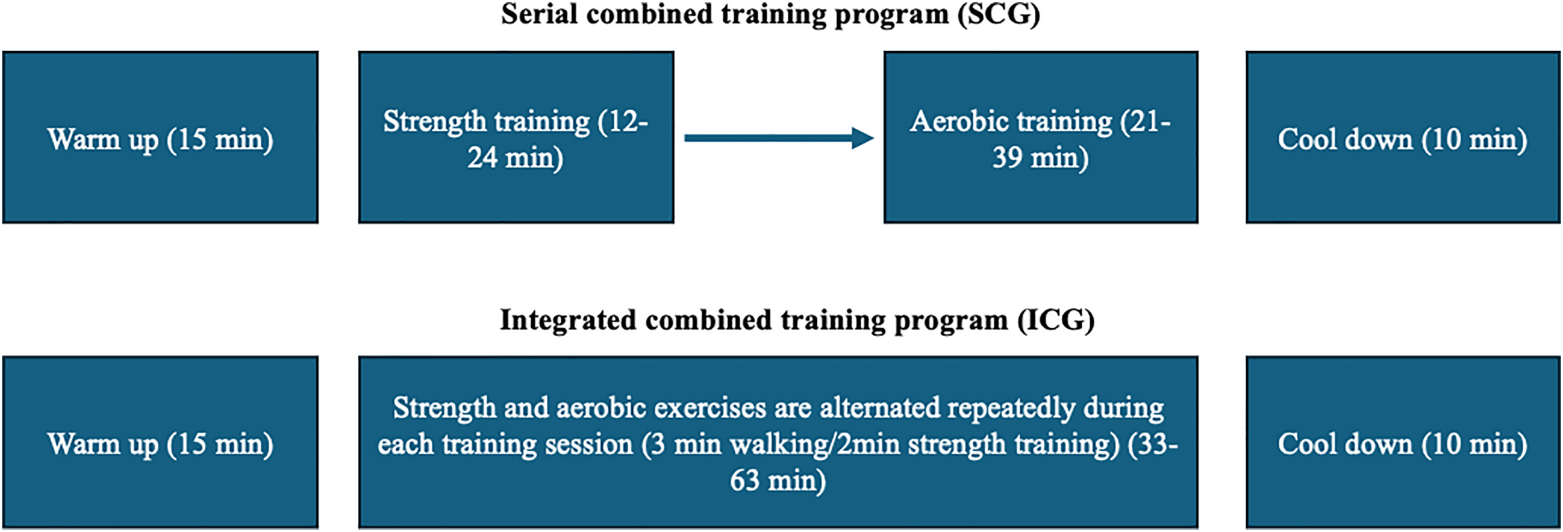
Schematic representation of the training protocols. Both groups performed a 15 min warm-up and a 10 min cool-down. In SCG, strength training was performed before aerobic training, whereas in ICG, strength and aerobic exercises were alternated (3 min walking/2 min strength training) throughout the session. Both groups performed the same aerobic and strength exercises during the workouts, using equivalent intensity, duration, volume, and training frequency.

Both groups participated in a 3-month combined training program (3 times/week). The duration of each training session was 58–88 min and consisted of 15 min of warm-up (10 min walking and 5 min stretching exercises), followed by 33–63 min of SCG or ICG. Each session was completed with a 10 min cool down period (5 min walking and 5 min stretching exercises). Both groups performed the same aerobic and strength exercises during the workouts, using equivalent intensity, duration, volume, and training frequency. Training intensity was controlled using an HR monitor, maintaining a target heart rate of 65%–80% HRmax throughout the training session. This intensity range was chosen based on established guidelines for improving cardiovascular fitness and metabolic health in untrained middle-aged individuals (1, 29). Thus, the only difference between the two groups was the sequence of aerobic and strength training. In SCG, the strength training was performed before aerobic training, while in ICG the aerobic and the strength training were alternated repeatedly in a predetermined order (3 min of walking/2 min of strength training) as described previously (1) (Figure 1).

The aerobic training program, for both exercise groups, consisted of walking at an intensity corresponding to 65%–80% of age-predicted HRmax (Polar Electro, Kempele, Finland) for 21–39 min. The intensity and duration of training progressively increased during the training program according to the procedures described previously (1). The strength training program included five resistance exercises targeting all major muscle groups: sit-ups, dorsals, push-ups, tricep dips, and lunges. Exercise intensity was progressively increased by adjusting the number of repetitions and sets throughout the intervention (see Table 2). Specifically, participants started with 2 sets of 8 repetitions per exercise and gradually progressed to 4 sets of 12 repetitions over the training period. This progression was implemented following established protocols (1) (see Table 2) to ensure safe and effective adaptation in untrained middle aged individuals. All training sessions were supervised by the project staff to ensure completion and adherence to the training program.

**Table 2:** Gradual increase of loading parameters during the serial and integrated combined training programs.

	Weeks			
	1–3	4–5	6–8	9–12
Serial combined training				
Aerobic training (walking)				
Intensity (% HR_max_)	65%–72%	70%–75%	70%–80%	75%–80%
Duration (min)	21	30	30	39
Strength training (body weight exercises)				
Sets	2	3	3	4
Reps/set	8–10	10	12	12
Rest time/set (s)	40–60	40–60	40–60	40–60
Integrated combined training				
Aerobic training (walking)				
Intensity (% HR_max_)	64%–73%	70%–76%	70%–80%	74%–80%
Duration (set × time)	7 × 3 min	10 × 3 min	10 × 3 min	13 × 3 min
Strength training (body weight exercises)				
Sets	2	3	3	4
Reps/set	8–10	10	12	12
Rest time/set (s)	Non-applicable			

% HRmax, percentage of the age-predicted maximum heart rate as recorded during the training program by the Polar V800 heart rate monitor.

### 2.4 Testing procedures

Before and after the completion of the 3-month training program, different health indices were measured.

### 2.5 Health indices

All health-related measurements were conducted before and after the 3-month intervention following a standardized protocol to ensure comparability. To minimize learning effects and measurement variability, participants first attended a familiarization session, where they were introduced to all assessment procedures and instructed on the correct execution on the tests. The testing process was explained in detail, and participants had the opportunity to practice certain measurements to ensure proper technique and consistency before data collection. Testing was conducted over two consecutive days. On the first day, anthropometric and body composition measurements were performed. During anthropometric measurements, participants stood fully upright, looking straight ahead, with heels together, barefoot, and with minimal clothing. Body height was measured using a physical beam scale (Seca model 220, Seca, Hamburg, Germany), with participants standing fully erect, looking straight ahead, and their heels together. Body mass was recorded with the same scale, ensuring participants remained still while standing. Body composition was assessed using bioelectrical impedance analysis (Maltron 900). Participants were instructed to avoid food, beverages, and exercise for at least three hours before the test and to urinate before the measurement to minimize fluid-related variability. They stood barefoot on the analyzer with their hands placed on the designated electrodes while maintaining a neutral posture. Waist-to-hip ratio was measured following WHO guidelines. Waist circumference was taken at the midpoint between the iliac crest and the inferior border of the last rib at the end of a normal exhalation. Hip circumference was measured at the widest part of the buttocks while ensuring the measuring tape was horizontal and parallel to the ground. All measurements were performed with a precision of 0.1 cm and repeated twice to ensure accuracy (35).

On the second day, cardiovascular, pulmonary, and metabolic assessments were conducted. Blood pressure was measured using an electronic blood pressure monitor (A&D-UA-851) after participants remained seated and at rest for 5 min in a quiet environment. The measurement was taken from the participant's dominant arm, positioned at heart level, and was repeated after 1 min to confirm consistency. Pulmonary function was assessed using spirometry (Micro Medical Micro). Participants were instructed to take a deep breath and then exhale forcefully into the spirometer while wearing a nose clip to prevent nasal airflow. Three trials were performed for each participant, and the highest values of forced vital capacity (FVC) and forced expiratory volume in one second (FEV_1_) were recorded. Total energy expenditure and macronutrient composition was calculated using sport watch Polar V800 (25, 36). Resting metabolic rate (RMR) was calculated using the Katch-McArdle formula, which is based on the fat free mass (FFM): RMR = 370 + (21.6 × FFM (kg)) (37). The total daily energy expenditure (TDEE) was calculated using the Katch-McArdle equation to determine RMR and adjusted for physical activity using an activity factor. The activity factor was determined based on participants' reported physical activity levels, with multipliers ranging from 1.2 (sedentary) to 1.9 (very active). TEE = RMR × activity multiplier (38).

To maintain consistency, all measurements were conducted by the same trained technician, who provided real-time feedback, ensured proper execution, and corrected any errors in participant performance. Before testing, participants attended a familiarization session where they received step-by-step verbal instructions and live demonstrations for each assessment. This session allowed them to practice specific tests under supervision to ensure proper technique and compliance with standardized procedures. Post-intervention assessments followed the same standardized protocols as pre-testing to allow for direct comparisons and minimize variability.

### 2.6 Statistical analysis

Results are presented as means ± standard deviations. Data were analyzed with IBM SPSS Statistics v.23 software. The normality of data was examined using the Shapiro–Wilk test, while homogeneity of variance was examined with Levene's test. 2-way ANOVAs (group × time; 2 × 2) with repeated measures on the “time” factor were used to analyze the data. Sidak pairwise comparisons were applied to locate the significantly different means within and between groups. One-way ANOVAs were used between groups to compare the relative changes from pre- to post-training. The magnitude of the difference was assessed by Hedges' g (*g*). The magnitude of the difference was considered small (0.2 < *g* ≤ 0.5), moderate (0.5 < *g* ≤ 0.8), or large (*g* > 0.8). Statistical significance was set at *p* < 0.05 for all analyses.

## 3 Results

**Figure 2:**
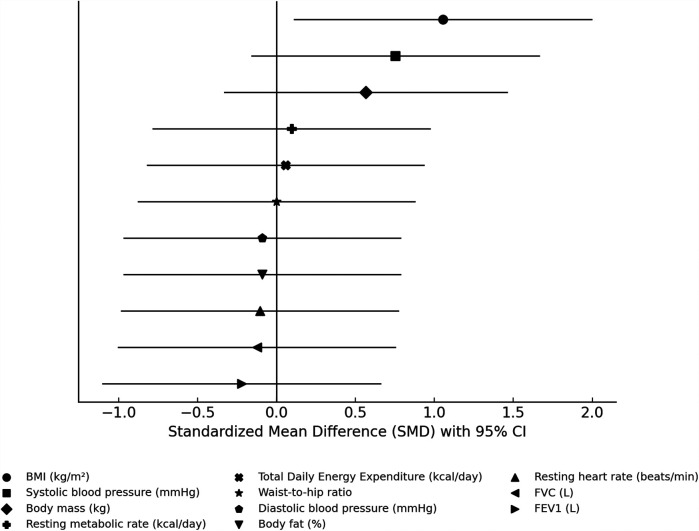
Standardized mean differences (SMDs) and 95% confidence intervals for the effects of SCG vs. ICG on physical health indices.

ANOVAs showed significant “group × time” interaction effects on health indices (*p* < 0.005, Table 3). Compared to pre-training values, after a 3-month training period (post-training) both groups (SCG and ICG) significantly (*p* < 0.005, *g* = 0.40–2.71) improved all health indices (Figure 2). More specifically, found a moderate decrease in %BF, a small decrease in body circumferences, and a moderate—large decrease in arterial blood pressure and resting heart rate as well as a large increase in FVC and FEV_1_ were observed. Furthermore, RMR significantly decreased (*p* < 0.005), while TDEE significantly increased (*p* < 0.005) (Table 4). Macronutrient composition also showed significant increases, with higher consumption of protein, carbohydrate, and fat (*p* < 0.005) (Table 5). There were no significant differences between SCG and ICG in any of the measurements (*p* > 0.05). Additionally, no significant differences were observed in average energy expenditure between SCG and ICG after training sessions (*p* > 0.05) (Table 6).

**Table 3:** Health indices in the two groups (SCG and ICG) before and after training sessions (values are means ± SD).

Variables	Groups	Pre-training	Post-training	Delta difference (90% CI)	Chances of effect	Qualitative assessment
Body mass (kg)	SCG	99.10 ± 21.23	91.47 ± 20.16	−7.63 (−9.08, −6.18)	Better	Significant weight loss, beneficial for reducing cardiometabolic disease risk
	ICG	96.20 ± 18.02	93.50 ± 17.95	−2.70 (−3.85, −1.55)	Better	Moderate weight loss, beneficial but less pronounced than in SCG
BMI (kg/m^2^)	SCG	31.27 ± 5.31	28.98 ± 4.62	−2.29 (−3.10, −1.48)	Better	Improvement in body composition, reducing obesity risk
	ICG	30.88 ± 4.69	30.01 ± 4.67	−0.87 (−1.52, −0.22)	Trivial	Small change, not clinically meaningful
Body fat (%)	SCG	30.77 ± 3.41	28.54 ± 2.75*	−2.23 (−3.01, −1.45)	Better	Effective fat reduction, important for metabolic health
	ICG	31.20 ± 4.78	28.15 ± 5.15*	3.05 (−4.38, −1.72)	Better	Substantial fat loss, potentially more effective than SCG
Waist-to-hip ratio	SCG	0.99 ± 0.05	0.97 ± 0.05*	−0.02 (−0.03, −0.01)	Better	Reduction in central obesity, associated with improved metabolic health
	ICG	0.98 ± 0.02	0.97 ± 0.02*	−0.01 (−0.02, 0)	Trivial	Minimal change, unlikely to impact health significantly
Systolic blood pressure (mmHg)	SCG	122.10 ± 5.97	118.65 ± 5.70*	−3.45 (−5.12, −1.78)	Better	Reduction in blood pressure, beneficial for cardiovascular health
	ICG	124.25 ± 4.34	119.25 ± 3.61*	−5.00 (−6.98, −3.02)	Better	Even greater reduction, important for hypertensive individuals
Diastolic blood pressure (mmHg)	SCG	80.70 ± 4.95	75.70 ± 4.06*	−5.00 (−6.74, −3.26)	Better	Improved blood pressure control, beneficial for heart health
	ICG	78.70 ± 2.61	75.50 ± 2.80*	−3.20 (−4.55, −1.85)	Better	Moderate reduction, supportive of cardiovascular health
FVC (L)	SCG	4.61 ± 0.19	4.76 ± 0.22*	0.15 (0.08, 0.22)	Better	Improvement in lung capacity, beneficial for respiratory function
	ICG	4.49 ± 0.06	4.64 ± 0.05*	0.15 (0.09, 0.21)	Better	Similar improvement, suggesting enhanced pulmonary efficiency
FEV_1_ (L)	SCG	3.91 ± 0.11	4.03 ± 0.11*	0.12 (0.06, 0.18)	Better	Improved respiratory function, beneficial for aerobic capacity
	ICG	3.81 ± 0.05	3.94 ± 0.05*	0.13 (0.07, 0.19)	Better	Comparable improvement, indicating increased lung function
Resting heart rate (beats/min)	SCG	73.60 ± 3.71	71.60 ± 3.56*	−2.00 (−3.44, −0.56)	Better	Reduction in resting heart rate, indicating improved cardiovascular fitness
	ICG	73.30 ± 4.99	69.30 ± 4.54*	−4.00 (−5.98, −2.02)	Better	Greater reduction, suggesting enhanced heart efficiency

* *p* < 0.005 pre-training vs. post-training in SCG and ICG.

SCG, serial combined group; ICG, integrated combined group; FVC, forced vital capacity; FEV_1_, forced expiratory volume in 1 s.

**Table 4:** Resting metabolic rate and total daily energy expenditure in the two groups (SCG and ICG) before and after training sessions (values are means ± SD).

Variables	Groups	Pre-training	Post-training	Delta difference (90% CI)	Changes of effect	Qualitative assessment
RMR (kcal/day)	SCG	1,853.5 ± 216	1,777.2 ± 205.6*	−76.3 (−86.3, −66.3)	Better	Decrease in RMR, expected as fat-free mass is a major determinant of RMR
	ICG	1,822 ± 182.54	1,795 ± 181.78*	−27.0 (−37.0, −17.0)	Trivial	Small reduction in RMR, likely due to metabolic adaptation
TDEE (kcal/day)	SCG	2,224.2 ± 259.2	2,754.66 ± 318.68*	530.46 (520.46, 540.46)	Better	Significant increase in TDEE, suggesting improved metabolic activity
	ICG	2,154 ± 218.14	2,782.25 ± 281.76*	628.25 (618.25, 638.25)	Better	Greater increase in TDEE, possibly due to enhanced physical activity levels

* *p* < 0.005 pre-training vs post-training in SCG and ICG.

RMR, resting metabolic rate; TDEE, total daily energy expenditure.

**Table 5:** Macronutrient composition in the two groups (SCG and ICG) before and after training sessions (values are means ± SD).

Variables	Groups	Pre-training	Post-training
Protein (g)	SCG	27.80 ± 3.24	34.43 ± 3.98*
	ICG	22.78 ± 2.28	34.78 ± 3.52*
Carbohydrate (g)	SCG	194.62 ± 22.68	241.03 ± 27.74*
	ICG	159.43 ± 15.96	243.45 ± 24.63*
Fat (g)	SCG	148.28 ± 17.28	183.64 ± 21.28*
	ICG	121.47 ± 12.17	185.48 ± 18.78*

* *p* < 0.005 pre-training vs. post-training in SCG and ICG.

**Table 6:** Average energy expenditure in the two groups (SCG and ICG) after training sessions (values are means ± SD).

Variable	Group	After training sessions	SMD (95% CI)
Average energy expenditure (kcal/day)	SCG	203.44 ± 76.12	0.23 (−0.65, 1.10)
	ICG	189.43 ± 43.85	

SCG, serial combined group; ICG, integrated combined group; SMD, Standardized mean difference; CI, Confidence interval.

## 4 Discussion

The purpose of this study was to examine and compare the energy expenditure, body composition, and metabolic rate between SCG and ICG in untrained middle-aged obese males. The main finding of this study was that there were no significant differences in average energy expenditure, resting metabolic rate, total energy expenditure and macronutrient composition between SCG and ICG after a 3-month combined training program.

This is the first study that compared the energy expenditure of SCG and ICG. However, several studies have examined the energy expenditure after the SCG. The study by Hunter et al. (22) found that 3 days/week of aerobic training and 3 day/week of resistance training significantly decreased energy expenditure by 150 kcal/day in untrained women aged 60–74 years. Dolezal and Potteiger (39) reported that resistance training and combined training groups (endurance and resistance training) showed significant increases in resting metabolic rate compared to the endurance training group.

Furthermore, only a few studies have examined the energy expenditure after the ICG. In a study by Monteiro et al. (24), compared the energy expenditure of circuit weight training with that of compounded circuit training (an integrated training program). The ICG induced higher energy expenditure than the typical circuit training.

Other important findings of the present study include that both training regimes significantly improved resting metabolic rate, with no significant differences observed between the two groups (SCG and ICG). This reduction in resting metabolic rate observed after a 3-month combined training program is a physiological adaptation, as fat-free mass is the main determinant of resting metabolic rate. Since body fat decreased, a concurrent reduction in resting metabolic rate was noted. The aligns with findings from Byrne and Wilmore (40), who reported a significant decrease in resting metabolic rate following 20 weeks of combined resistance and aerobic training (walking). This decline in RMR may be attributed to metabolic adaptation, a well-documented physiological response to weight loss and prolonged energy deficit. Specifically, reductions in body mass and fat stores often lead to compensatory decreases in energy expenditure, as the body attempts to preserve energy balance by downregulating thermogenesis and hormonal activity (e.g., leptin and thyroid hormones) (41, 42). These findings are consistent with previous research demonstrating that sustained weight loss and increased physical activity can induce a proportional decline in RMR, potentially as a survival mechanism to counteract energy deficits (43).

In this study, significantly improved total daily energy expenditure, with no significant differences between the two groups. The increase in total daily energy expenditure highlights the effectiveness of combined training programs in boosting daily energy expenditure, which is crucial for weight managements and improving metabolic health. These results align with prior studies indicating that exercise programs combining resistance and aerobic components significantly elevate total daily caloric expenditure (22). For example, the study by Hunter et al. (22) found that total daily energy expenditure increased after 16 weeks of combined aerobic and resistance training in older women.

Both groups demonstrated significant increases in macronutrient intake after the training period, with no significant differences between groups. These findings highlight the role of dietary adaptations in optimizing training outcomes, ensuring energy availability, and supporting metabolic health, while also aligning with previous literature emphasizing the importance of macronutrient balance in exercise performance and recovery. Macronutrient intake was estimated using a combination of smartwatch-based tracking and self-reported food records. While this approach provides insights into dietary patterns, it is important to acknowledge potential limitations, including variability in wearable-based estimations and potential recall inaccuracies in self-reported data. These factors should be considered when interpreting the impact of dietary adaptations on training outcomes (44, 45).

Other important findings of the present study were that both training regimes decreased body fat, body waist-to-hip ratio, and blood pressure, while increasing respiratory function. Our findings for the SCG aerobic (walking) and strength training (body weight strength exercises) program are in line with finding from previous studies reporting improvements in body fat, and body weight in middle-aged men following SCG (1, 11–15). Other studies, however, did not observed positive training adaptations in body weight, BMI, and waist circumference (13–15). The differences observed between the results from the earlier studies and the present study could be attribute to variations in subjects' characteristics and training loads. However, the primary factor is likely the order of exercises, which may amplify the interference effect and reduce the efficacy of SCG strength and aerobic training programs in improving neuromuscular performance (46, 47).

Furthermore, there are limited studies on ICG for middle-aged men. Previous studies showed similar improvements in body fat, body weight, and waist circumference in middle-aged men (1, 48–50). However, some of the previous studies have found no improvements in body fat, body weight, and waist circumference (48, 49). These findings highlight the need for careful consideration of exercise sequencing in combined training programs, as the interference effect may influence neuromuscular adaptations. Given the mixed results in body composition outcomes, exercise prescription for obese populations should emphasize individualized approaches, progressive overload, and long-term adherence strategies to maximize metabolic benefits. The discrepancies between the results of previous studies and the present study could be attributed to differences in aerobic training modalities, subject characteristics, loading parameters, and training frequency.

### 4.1 Limitations

A key limitation of this study is the reliance on predictive equations to estimate resting metabolic rate, total daily energy expenditure, and macronutrient intake. While these equations are widely used and validated for general populations, they may not fully account for individual metabolic variability, particularly in untrained, middle-aged obese males. Additionally, the study relied on self-reported dietary intake, which is subject to recall bias and potential underreporting, further impacting the accuracy of macronutrient estimations. Another limitation is the absence of a control group, which restricts the ability to directly attribute observed changes to the intervention. The relatively small sample size also limits the generalizability of the findings.

Future research should aim to improve measurement accuracy by incorporating gold-standard methods such as indirect calorimetry and doubly labeled water for metabolic assessments. The inclusion of direct calorimetry could provide an even more precise evaluation of energy expenditure, further refining predictive models used in metabolic research. Additionally, future studies should consider using objective dietary tracking tools, such as wearable devices or biomarkers, to minimize biases associated with self-reported intake and enhance measurement accuracy.

## 5 Conclusion

This study compared the effects of SCG and ICG on energy expenditure, body composition, and metabolic rate in untrained middle-aged obese males. The findings demonstrated no significant differences between the two training modalities in energy expenditure, resting metabolic rate, or macronutrient intake after three months. Both programs were effective in increasing total daily energy expenditure, optimizing macronutrient oxidation, and supporting metabolic health. In addition, both training regimens significantly decreased body fat, waist-to-hip ratio, and blood pressure while enhancing respiratory function. These results highlight the effectiveness of combined training in improving key health markers. Differences between this study and prior research may reflect variations in participants' characteristics, training protocols, and exercise modalities. Overall, both SCG and ICG appear to be viable strategies for promoting metabolic health and improving body composition in middle-aged obese males. Future research should focus on long-term outcomes, including training sustainability, exercise adherence, and metabolic adaptations, to further refine and optimize training programs for this population.

## Data Availability

The raw data supporting the conclusions of this article will be made available by the author, without undue reservation.
